# Cardiopulmonary coupling indices to assess weaning readiness from mechanical ventilation

**DOI:** 10.1038/s41598-021-95282-2

**Published:** 2021-08-06

**Authors:** Pablo Armañac-Julián, David Hernando, Jesús Lázaro, Candelaria de Haro, Rudys Magrans, John Morales, Jonathan Moeyersons, Leonardo Sarlabous, Josefina López-Aguilar, Carles Subirà, Rafael Fernández, Michele Orini, Pablo Laguna, Carolina Varon, Eduardo Gil, Raquel Bailón, Lluís Blanch

**Affiliations:** 1 Biomedical Signal Interpretation and Computational Simulation (BSICoS) group at the Aragón Institute of Engineering Research (I3A), IIS Aragón, University of Zaragoza, Zaragoza, Spain; 2 CIBER de Bioingeniería, Biomateriales y Nanomedicina (CIBER-BBN), Instituto de Salud Carlos III, Madrid, Spain; 3 Critical Care Center, Hospital Universitari Parc Taulí, Institut d’Investigació Parc Taulí I3PT, Universitat Autónoma de Barcelona, Sabadell, Spain; 4 CIBER de Enfermedades Respiratorias (CIBER-ES), Instituto de Salud Carlos III, Madrid, Spain; 5 Better Care, Barcelona, Spain; 6 Department of Electrical Engineering-ESAT, STADIUS Center for Dynamical Systems, Signal Processing and Data Analytics, KU Leuven, Leuven, Belgium; 7 Department of Intensive Care, Fundació Althaia, Universitat Internacional de Catalunya, Manresa, Spain; 8 Institute of Cardiovascular Science, University College London, London, UK; 9 Barts Heart Centre, St Bartholomews Hospital, University College London, London, UK; 10 Circuits and Systems (CAS) group, Delft University of Technology, Delft, The Netherlands

**Keywords:** Biomedical engineering, Predictive markers, Predictive markers, Respiration, Blood flow, Scientific data

## Abstract

The ideal moment to withdraw respiratory supply of patients under Mechanical Ventilation at Intensive Care Units (ICU), is not easy to be determined for clinicians. Although the Spontaneous Breathing Trial (SBT) provides a measure of the patients’ readiness, there is still around 15–20% of predictive failure rate. This work is a proof of concept focused on adding new value to the prediction of the weaning outcome. Heart Rate Variability (HRV) and Cardiopulmonary Coupling (CPC) methods are evaluated as new complementary estimates to assess weaning readiness. The CPC is related to how the mechanisms regulating respiration and cardiac pumping are working simultaneously, and it is defined from HRV in combination with respiratory information. Three different techniques are used to estimate the CPC, including Time-Frequency Coherence, Dynamic Mutual Information and Orthogonal Subspace Projections. The cohort study includes 22 patients in pressure support ventilation, ready to undergo the SBT, analysed in the 24 h previous to the SBT. Of these, 13 had a successful weaning and 9 failed the SBT or needed reintubation –being both considered as failed weaning. Results illustrate that traditional variables such as heart rate, respiratory frequency, and the parameters derived from HRV do not differ in patients with successful or failed weaning. Results revealed that HRV parameters can vary considerably depending on the time at which they are measured. This fact could be attributed to circadian rhythms, having a strong influence on HRV values. On the contrary, significant statistical differences are found in the proposed CPC parameters when comparing the values of the two groups, and throughout the whole recordings. In addition, differences are greater at night, probably because patients with failed weaning might be experiencing more respiratory episodes, e.g. apneas during the night, which is directly related to a reduced respiratory sinus arrhythmia. Therefore, results suggest that the traditional measures could be used in combination with the proposed CPC biomarkers to improve weaning readiness.

## Introduction

Most patients admitted to Intensive Care Units (ICU) present symptoms of respiratory failure and need the support of Mechanical Ventilation (MV). MV is a procedure of artificial respiration that supplies or collaborates with a patient’s respiratory function, in order to have an efficient gas exchange and reduce the respiratory effort. As the respiratory muscles and the nervous system recover, patients are prepared to maintain normal breathing autonomously, making the respiratory support unnecessary. This process of withdrawing MV is known as “Weaning” and is a challenging and very delicate procedure. The American Thoracic Society (ATS) and the American College of Chest Physicians (CHEST) identify weaning as an investigation priority^1^. The international consensus conference in 2007^2^, recommends that weaning should be considered as soon as possible. However, weaning failure and reintubation due to premature liberation can lead to an increased risk of severe respiratory complications causing dyspnoea and hypoxaemia, such as ventilator induced lung injury (VILI)^3^, ventilator-associated pneumonia (VAP)^4^, or ventilator induced diaphragmatic dysfunction^5^. These complications are significantly associated with increased mortality, reported to range from 25–50%^6–8^.

Once a patient is deemed ready for weaning^9–11^, the Spontaneous Breathing Trial (SBT) assesses their ability to breathe independently^2^. It is carried out for nearly 30 min, by a low-level inspiratory pressure support –with minimal MV support– or a T-tube test –with no MV support^9–11^. Currently, the SBT is the best diagnostic test to determine more accurately if the weaning attempt will be successful or not. However, literature shows that about 20% of patients fail the SBT or, even worse, pass the SBT but do not respond to weaning, needing reintubation within the next 48 hours^8^. This causes patient suffering and abruptly increases the risk of complications and comorbidities^12,13^. Therefore, there is a need to improve weaning readiness criteria in order to reduce the number of patients undergoing SBT who are not ready to breathe spontaneously.

Recent clinical and electrophysiological studies revealed a high incidence of Autonomic Nervous System (ANS) dysfunction in patients admitted to the ICU^14,15^, which has a negative impact on clinical prognosis and mortality rates^16^. The ANS is responsible for cardiovascular control between heart, lungs and brain, to dynamically couple and regulate blood pressure, HR and respiration^17,18^. Therefore, some works studied the ANS recovery in order to better define the SBT outcome. Heart rate variability (HRV) is a non-invasive measurement of the ANS function, and it has been proven as a tool to evaluate the SBT outcome. Patients who failed SBT were observed to have reduced HRV during SBT compared to after weaning^19,20^. The study in^21^ demonstrates the added value of HRV and respiration rate variability, comparing the SBT outcome in the 30 min during the SBT. They found that altered HRV and respiration rate variability during the SBT are associated with weaning failure. Other works also found that comparing the HRV parameters before, during and after the SBT, the decision of weaning improved^22,23^. Based on these previous observational studies, in which HRV and respiratory parameters during SBT have been shown to differ between success and failure of SBT, we wanted to investigate if ANS dynamics could provide valuable information for weaning success prediction, before the SBT.

In addition to HRV, another important mechanism known as Cardiopulmonary Coupling (CPC) can be derived if HRV is combined with respiratory information^24^. The CPC is mainly expressed through the Respiratory Sinus Arrhythmia (RSA), which is the acceleration and deceleration of the heart driven by respiration^25^. The physiological role of the RSA is still a matter of debate, but it is widely accepted that RSA helps to match perfusion and ventilation during the respiratory cycle, enhancing pulmonary gas exchange and improving cardiac efficiency^25,26^. Correspondingly, although a lot of discussion exists on which mechanism is responsible for RSA^27–29^, studies suggest that the ANS, via the vagus nerve, plays the major role in it, together with some mechanical effects and the baroreflex. In fact, despite the limited understanding of the RSA function, some studies found the CPC as an effective ambulatory biomarker of sleep quality^30,31^, predictor for early treatment response in depressed patients^32^, or for asthma evolution monitoring in children^33^. Therefore, in this work, it is hypothesized that estimators of the CPC could also respond to the multi-factorial nature of the weaning process.

Different methods exist to quantify the strength of the RSA for CPC assessment, among which the following stand out^34^: analysis of the HRV in the High Frequency (HF) band^35^, Time Frequency Coherence (TFC)^36^, Information Dynamics (ID)^37^, Orthogonal Subspace Projections (OSP)^38^, Bivariate Phase Rectified Signal Averaging^39^, Pole Specific Spectral Causality^40^, and Cardiopulmonary Phase Synchronization^41^. The performance of these estimators of CPC is studied in^34^, and the indices derived from TFC, ID and OSP outperformed the others, which are the ones also used in this work.

This study is a proof of concept, aimed at finding new biomarkers that can help clinicians better predict whether a patient is ready to be weaned. As a matter of fact, no previous work investigated the heart-lung interactions or studied the usefulness of CPC indices in this context. The essential and fundamental innovations of this study are also the prospective design and that the measurements are blind to the process of SBT. Then, differences are sought only the 24 h before SBT, pursuing an improvement in the prediction of weaning readiness. The present paper evaluates the reliability of HRV and, especially, of CPC estimators as predictors of weaning outcome. Finally, the influence of the timing of the analysis is also evaluated, both throughout the day and in relation to the timing of SBT.

## Materials

### Database

The database was constructed prospectively at two hospitals in Spain, in the *Hospital Universitari Parc Taulí* and the *Fundació Althaia*, using the connectivity platform Better Care (Better Care, Barcelona, Spain. US patent No. 12/538,940).

This database was aimed to establish a new model for the prediction of successful weaning (ClinicalTrial.gov, NCT03451461). The Institutional Review Boards of *Comitè d’Ética d’Investigació amb medicaments* at the *Corporació Sanitària Parc Taulí* and the *Clinical Research Ethics Committee* of *Fundació Unió Catalana d’Hospitals* approved the database and the study protocol. The requirement for informed consent was waived as part of the study approval, since the current study was an ancillary analysis. Therefore, all the signals were anonymous and encrypted to ensure privacy. The guidelines followed in this study were according to the applicable Spanish regulations (Biomedical Research Law 14/2007).

The original dataset included 60 patients screened at the time of implementing the present study. Patients with neurological disorder, dementia or focal brain injury at ICU admission were excluded. Patients with arrhythmias, such as atrial fibrillation, were also excluded from the analysis, since HRV cannot be used as an estimate of the ANS function. In addition, only those patients ventilated with assist/support ventilation modes were considered, i.e., excluding patients that were in controlled ventilation modes in the 24 h previous to the SBT.

**Figure 1:**
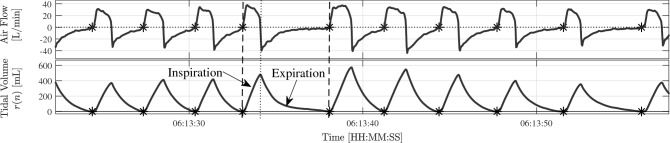
Example of respiration in PSV mode. The airflow signal is plotted at (top) and the derived tidal volume signal at (bottom). The onset of inspiration, delineated by Better Care, is marked with asterisks.

Then, the study cohort consists of 22 patients with heterogeneous clinical pictures, and data during the 24 h prior to the SBT were obtained. After SBT, patients were distributed into two groups, those with successful weaning and those without. For this subset, the most common MV mode was Pressure Support Ventilation (PSV), but also some patients spent some time in Continuous Positive Airway Pressure (CPAP). An example of the respiratory pattern in PSV mode is shown in Fig. 1.

### Patient classification and demographics

**Figure 2:**
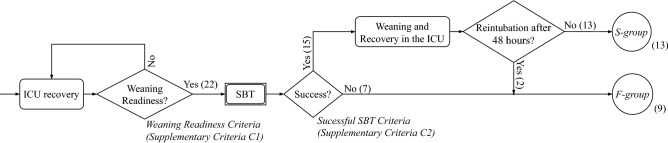
Algorithm for the definition of the weaning success. Patients are classified into the *S-group* or *F-group* after the Spontaneous Breathing Trial (SBT). The *S-group* stands for the group of patients successfully weaned (successful SBT and no need of reintubation). *F-group* stands for the group of patients with SBT failure and patients with SBT success but with the need of reintubation after 48 h of weaning. Numbers in parentheses represent the number of patients according to the respective criteria.

When the health condition of a patient improved enough, they are deemed ready for weaning (supplementary Criteria C1). However, at this time, these patients must perform the SBT. Patients presenting at least one item of the intolerance criteria for successful SBT (supplementary Criteria C2), were not ready for discontinuation and weaning failure was considered. These patients belong to the *F-group*. Patients who passed SBT, belong to the *S-group*. However, 2 patients that passed the SBT required orotracheal intubation or reconnection to non-invasive MV within 48 hours after SBT. These 2 patients were reclassified in the *F-group*. See Fig. 2 for the patient classification scheme.

With all these premises, there are 13 patients in the *S-group* and 9 in the *F-group*. According to the literature, for the analysed subset, actual weaning readiness is for the 54% of patients, and the SBT was incorrect for the 13% of the tests that passed.

The demographics of patients are summarized in Table 1. The variables available include: age, gender, Acute Physiology and Chronic Health Evaluation II (APACHE II), Sequential Organ Failure Assessment (SOFA), reason for MV, MV duration, ICU length of stay, ICU mortality and in-hospital mortality.

**Table 1:** Demographics. Data are presented as median [IQR 25–75] and percentages.

	S-group	F-group
	(N = 13)	(N = 9)
Age (years)	65 [60–72]	69 [59–72]
Gender (% female)	15%	33%
APACHE II at admission	18 [14–23]	16 [10–20]
SOFA at admission	7 [6–8]	6 [3–10]
Reason for MV :		
$\bullet$ Acute Respiratory Failure	30.7%	22.2%
$\bullet$ Sepsis	38.5%	33.3%
$\bullet$ Sepsis + ARDS	7.7%	11.1%
$\bullet$ Neurologic	15.4%	11.1%
$\bullet$ Cardio-Respiratory Arrest	–	11.1%
$\bullet$ Acute Pancreatitis	7.7%	11.1%
MV duration (days)	6 [4–10]	12 [8–16]
ICU length of stay (days)	8 [6–12]	18 [13–23]
ICU mortality	7.69%	22.22%
In-hospital mortality	7.69%	22.22%

APACHE: Acute Physiology and Chronic Health Evaluation; SOFA: Sequential Organ Failure Assessment; MV: Mechanical Ventilation; ICU: Intensive Care Unit. ARDS: Acute Respiratory Distress Syndrome

### Data acquisition and data analysis

Physiological signals were continuously recorded using the connectivity platform Better Care^42^. The Better Care system (Better Care, Barcelona, Spain. US patent No. 12/538,940), is a proprietary system for data collection designed to interact with output signals from mechanical ventilators and bedside monitors rather than directly with patients. It was firstly developed to interoperate signals from different ventilators and monitors, and subsequently compute algorithms for diagnosing patient-ventilator asynchronies.

Better Care standardizes, synchronizes and stores the signals of all the bedside monitors and ventilators at 200 samples per second, from intubation in the ICU to liberation from MV. Different biomedical signals were recorded, including the three bipolar leads of the electrocardiogram (ECG), as well as the respiratory signals: airflow and airway pressure. In addition, pulse photoplethysmography, blood pressure via invasive catheter, and SpO$_2$ were also recorded.

The onset of inspiration for each breath was delineated using the algorithms implemented in the Better Care platform. For each respiratory cycle, information on the type of ventilation mode, the trigger of respiration and the appearance or absence of asynchronies, such as ineffective efforts or double cycling, were also given^42,43^. It is possible that a patient is in PSV mode and the machine automatically triggers a breath, e.g. due to low respiratory rate. However, all breathing cycles are delineated by Better Care, considering the characteristic morphologies of the airway pressure and airflow^42^. Then, these automated breathing cycles are labelled as ‘controlled’ and they are omitted for the analysis of the respiratory signal.

## Methods

The methods are established using ECG and respiratory signal processing techniques. First, the tidal volume and HRV signals are estimated. Afterwards, three different techniques are used to compute the CPC indices, with the objective of obtaining an estimate of the RSA. These indices are computed based on TFC, ID and OSP. However, although these three CPC estimates rely on different techniques, the underlying idea is the same: to measure and characterize the RSA function, i.e., respiratory-circulatory interactions, in patients ready for weaning the 24 h before the SBT. Table 2 summarizes all the terms and indices computed in this work.

As mentioned, the algorithms do not extract the information in the same way, and the indices have different temporal resolution. For this reason, the average value in consecutive 30-min periods is considered for each index calculated in this work, in order to be able to compare all the indices through the 24 h recordings.

**Table 2:** Summary of terms and indices computed.

F$_r$	Respiratory Frequency	
HR	Heart Rate	
*HRV*	*Heart Rate Variability*	
SDNN	Standard Deviation of Normal-to-Normal intervals	
RMSSD	Root-Mean Square of Successive intervals Differences	
${\text{ P }}_{{\rm VLF}}$	Power in the VLF band	
${\text{ P }}_{{\rm HF}}$	Power in the modified HF band, centred to respiration	
${\text{ P }}_{{\rm LF}}^n$	Normalized Power ${\text{ P }}_{{\rm LF}}$/(${\text{ P }}_{{\rm LF}}$+${\text{ P }}_{{\rm HF}}$), with ${\text{ P }}_{{\rm HF}}$ centred to respiration	
*CPC*	*Cardio-Pulmonary Coupling*	Framework
$\mathscr {C}^{\mathcal {T}}_{{\rm HF}}$	Amount of spectral coherence between HRV and respiration, averaged in time where coherence is significant	TFC Time-Frequency Coherence
$\mathscr {CE}_{{\rm r} \leftrightarrow {\mathrm{m}}}$	Cross entropy, amount of information shared between HRV and respiration	ID Information Dynamics
${\mathscr {P}}_{{\rm m}_{{\rm r}}}$	Relative power of the respiratory component embedded in HRV	OSP Orthogonal Subspace Projections

### HRV and respiratory information estimation

The baseline-corrected tidal volume signal, *r*(*t*), is obtained integrating the instantaneous airflow signal followed by baseline subtraction. The baseline is estimated by modified Akima piecewise cubic Hermite interpolation at the onsets of inspiration. This ensures that each tidal volume breath begins and ends with zero litres, as depicted in Fig. 1. The respiratory frequency signal, $F_r(t)$, is derived from the estimation in each breath by computing the inverse of the instantaneous inspiration-to-inspiration difference, F$_r$. After that, *r*(*t*) and $F_r(t)$ are resampled at 4 Hz, to obtain the tidal volume signal, *r*(*n*), and the respiratory frequency signal, $F_r(n)$, respectively.

The lead II of the ECG is upsampled at 1000 Hz with cubic spline interpolation, to ensure that HRV analysis is not compromised by the effect of low sampling frequency^35^. Then, the QRS-complexes are detected by means of a wavelet-based method^44^. The time between two successive R waves defines the RR interval. Ectopic beats and miss-detections are corrected as described in^45^. The exclusion of non-normal RR intervals, which do not represent the ANS function, results in the normal-to-normal (NN) interval series. The temporal indices of HRV are calculated from the NN series^35^: Standard Deviation of the NN intervals (SDNN), and Root Mean-Square of Successive Differences of adjacent NN intervals (RMSSD).

For frequency domain analysis, the HRV signal is estimated using the Time-Varying Integral Pulse Frequency Modulation model (TVIPFM)^46^. The resulting modulating signal, *m*(*t*), is assumed to contain the ANS modulation of the sinoatrial node. Essentially, given a particular beat time occurrence, *m*(*t*) is defined as: $m(t) = ( d_{{{{\rm HR}}}}(t)-d_{{{{\rm HRM}}}}(t))/d_{{{{\rm HRM}}}}(t)$, where $d_{{{{\rm HR}}}}(t)$ represents the instantaneous HR signal obtained from the TVIPFM model, and $d_{{{{\rm HRM}}}}(t)$ is obtained by low-pass filtering $d_{{{{\rm HR}}}}(t)$ at 0.03 Hz. Finally, the evenly sampled version of the modulating signal, *m*(*n*), is obtained by resampling *m*(*t*) at 4 Hz.

Previous studies found that HRV analysis guided by respiration improved the ability of HRV to discriminate cognitive stress in healthy subjects^47,48^. In the same way in this work, the HF band is redefined to be time-varying and centred at the respiratory frequency: $\Omega _{{\rm HF}}^r (t)=[F_r(t)-0.15, F_r(t)+0.15]\,\hbox {Hz}$. HF power, ${\text{ P }}_{{\rm HF}}$, is defined as the power within this $\Omega _{{\rm HF}}^r$ band. The use of the modified HF band is also specifically encouraged by the increased F$_r$ observed in some mechanically ventilated patients, who had a respiratory frequency higher than 24 rpm, i.e., 0.4 Hz. Therefore, the need to center the HF band to the respiratory frequency is crucial to perform a proper and more powerful interpretation of the results in the frequency domain, in order to avoid an underestimation of the HF power using the classical HF band. Low Frequency (LF) power, ${\text{ P }}_{{\rm LF}}$, is defined as the power in the classic LF band^35^: $\Omega _{{\rm LF}}=[0.04,0.15]$ Hz.

The sympathovagal balance is represented by the power normalized in the LF band^35^: ${\text{ P }}_{{\rm LF}}^n = {\text{ P }}_{{\rm LF}} / ({\text{ P }}_{{\rm LF}} +{\text{ P }}_{{\rm HF}} )$. In addition, the Very Low Frequency (VLF) power, ${\text{ P }}_{{\rm VLF}}$, is also quantified since most of the power of HRV in 24 h recording resides in the frequencies below HF and LF power. The ${\text{ P }}_{{\rm VLF}}$ is accounted for by fluctuations in NN intervals that have a period larger than 25 s: $\Omega _{{\rm VLF}}=(0,0.04]$ Hz. The physiological role of ${\text{ P }}_{{\rm VLF}}$ is still not clear, but it is believed to be related to the circadian rhythms and core body temperature^49^. The frequency domain parameters are calculated using a Time-Frequency (TF) distribution belonging to the Cohen’s class^36^. A time and frequency resolution of 11.25 s and 0.039 Hz is used, respectively.

### Time-frequency coherence

The respiratory influences on HRV can be captured based on TFC, which is given by:

1 $$\begin{aligned} \hat{\gamma }(t,f) = \frac{\left| \hat{S}_{r,m}(t, f)\right| }{\sqrt{\hat{S}_{r}(t, f) \hat{S}_{m}(t, f)}}, \end{aligned}$$

where $\hat{\gamma }(t,f) \in [0,1]$. $\hat{S}_{r}(t,f)$ and $\hat{S}_{m}(t,f)$ are the auto-power spectral densities calculated by means of the Cohen’s Class Wigner Ville Distribution^36^ of respiration, here represented by its surrogate tidal volume *r*(*n*), and HRV, represented by *m*(*n*), respectively. $\hat{S}_{r,m}(t, f)$ is the cross-power spectral density. To obtain $\hat{S}_{r}(t, f)$, $\hat{S}_{m}(t,f)$ and $\hat{S}_{r,m}(t,f)$, the same TF maps are used as those used for the estimation of the HRV frequency domain parameters^36^ (see previous section). An illustrative example of the TFC performance can be seen in supplementary Figure F1.

A significant coherence level of coupling between HRV and respiration is set by the signal-independent threshold, $\gamma _{{\rm TH}}(t,f;\alpha )$. It is established based on a surrogate data analysis^36^, with $\alpha =5\%$ risk that both signals are coupled when real coupling does not exists $\gamma _{{\rm TH}}(t,f;0.05)=\gamma _{{\rm 0}}$. The region $\Omega _{{\rm HF}}^{r,c}(t,f)$, from which the coherence is estimated, is identified as the region where the TFC is significant within the HF band centred at the respiratory frequency, $\Omega _{{\rm HF}}^r (t)$:

2 $$\begin{aligned} \Omega _{{\rm HF}}^{r,c} (t,f) = \left\{ (t,f) \in (\mathbb {R}^{+} \times \Omega _{{\rm HF}}^r(t) )\ |\ \hat{\gamma }(t,f)>\gamma _{{\rm 0}}\right\} . \end{aligned}$$

To characterize the temporal evolution of the local coupling between the spectral components of the signals, the index $\mathcal {C}_{{\rm HF}}(t)$ is defined as:

3 $$\begin{aligned} \mathcal {C}_{{\rm HF}}(t)=\int _{\Omega _{{\rm HF}}^{r,c}} \hat{\gamma }(t, f) d f \biggr / \int _{\Omega _{{\rm HF}}^{r,c}} 1\ df . \end{aligned}$$

This index takes into account the magnitude of the local coupling, averaged in the HF band. Now, if the mean significant coherence, $\mathcal {C}_{{\rm HF}}(t)$, is averaged in a period of time, it yields to the definition of $\mathscr {C}_{{\rm HF}}$:

4 $$\begin{aligned} \mathscr {C}_{{\rm HF}} = \int \mathcal {C}_{{\rm HF}}(t) dt \biggr / \int 1\ dt . \end{aligned}$$

Finally, for all the time course, the existence of significant coupling at any frequency in the whole band $\Omega _{{\rm HF}}^r (t)$ is identified as:

5 $$\begin{aligned} \mathcal {T}_{{\rm HF}}(t)= \left\{ \begin{array}{ll} {1,} &{} {{\rm if}\ \Omega _{{\rm HF}}^{r,c} (t,f) \ne \emptyset \ } \\ {0,} &{} {{\rm if}\ \Omega _{{\rm HF}}^{r,c} (t,f) = \emptyset \ }\end{array}\right. \end{aligned}$$

Once the “mask” $\mathcal {T}_{{\rm HF}}(t)$ is defined, the percentage where TFC is significant in a period of time, $\mathscr {T}_{{\rm HF}}$, can be defined as:

6 $$\begin{aligned} \mathscr {T}_{{\rm HF}} = \int \mathcal {T}_{{\rm HF}}(t) dt \biggr / \int 1\ dt . \end{aligned}$$

The index of CPC used from this framework is calculated using Eqs. (3) and (5). This index is denoted as $\mathscr {C}^{\mathcal {T}}_{{\rm HF}}$ and is composed taking into account the mean significant coherence averaged in a period of time, $\mathscr {C}_{{\rm HF}}$, with the percentage of time where TFC is significant in that same period, $\mathscr {T}_{{\rm HF}}$:

7 $$\begin{aligned} \mathscr {C}^{\mathcal {T}}_{{\rm HF}} = \mathscr {C}_{{\rm HF}} \cdot \mathscr {T}_{{\rm HF}} . \end{aligned}$$

### Dynamic mutual information

It is known that the RSA defines a causal relationship from respiration to HRV, since respiration drives acceleration/deceleration in the HR. This relationship implies that the uncertainty about HRV, can be resolved not only by knowing itself, but also by taking into account the information transferred from respiration. This resolution of entropy, or uncertainty, can be quantified using measures of predictive information^37^.

Let’s denote $r_{n}$ and $m_{n}$ as the scalar random values obtained by sampling the process *r*(*n*) and *m*(*n*), respectively, at the present time, *n*. The vectors $\mathbf {r}^{-} = [ r(n-1), \ldots, r(n-M)]^{T}$, and $\mathbf {m}^{-} = [ m(n-1), \ldots, m(n-M)]^{T}$, are defined to describe the whole past of each process, with *M* the model order. If the information carried by the HRV is split into components related to respiration and others, the predictive information leads to the definition of the Cross Entropy term, $\mathscr {CE}_{{\rm r} \leftrightarrow {\mathrm{m}}}$. This term quantifies the amount of information shared at a certain time, *n*, between the present value of HRV, $m_{n}$, and the past of respiration, $\mathbf {r}^{-}$:

8 $$\begin{aligned} \mathscr {CE}_{{\rm r} \leftrightarrow {\mathrm{m}}} = I\left( m_{n} ; \mathbf {r}^{-} \right) = H\left( m_{n} \right) - H\left( m_{n} | \mathbf {r}^{-} \right) , \end{aligned}$$

where $I(\cdot ;\cdot )$ quantifies the *mutual information*, $H\left( m_{n} \right)$ expresses the amount of information carried by the process in terms of the average uncertainty about $m_{n}$, the so-called *Shannon entropy*. $H\left( m_{n} | \mathbf {r}^{-} \right)$ denotes the *conditional entropy* and it quantifies the average uncertainty that remains about $m_{n}$ when $\mathbf {r}^{-}$ is known^37^. The model order, *M*, is defined as the minimum amount of delays obtained using both the Minimum Description Length principle and the Akaike Information Criterion. The maximum possible delay is set to 10 s in order to avoid over-fitting. The minimum possible delay is set to the period equivalent to the lowest frequency of the respiration bandwidth in order to avoid a too-simple model.

### HRV decomposition

By using subspace projections, the HRV can be decomposed into two different components^38^. First, the component describing all variations of HRV linearly related to respiration is derived. After that, the remainder, namely residual component, describes all dynamics modulated by other mechanisms different from respiration, such as the sympathetic modulations or other vagal modulators unrelated to respiration, plus the possible non-linear influences of respiration.

Given are the respiratory signal, *r*(*n*), and the HRV estimated from the modulating signal, *m*(*n*). The vectors $\mathbf {r} = [ r(0), r(1), \ldots, r(N-M+1)]^{T}$ and $\mathbf {m} = [ m(0), m(1), \ldots, m(N-M+1) ]^{T}$ are defined to construct a respiratory subspace, with *N* the number of samples in a computational period and *M* the number of delays. The model order, *M*, is the same used for the $\mathscr {CE}_{{\rm r} \leftrightarrow {\mathrm{m}}}$ computation (see previous section). The OSP projects $\mathbf {m}$ onto the subspace $\mathbb {V}$, which is the subspace defined by all variations in $\mathbf {r}$. The matrix $\mathbf {V}$ spans the subspace $\mathbb {V}$, and it is constructed as a time-delay embedding of $\mathbf {r}$, using *M* delays. Once the matrix $\mathbf {V}$ is constructed, the HRV can be projected onto the respiratory subspace $\mathbb {V}$, by means of the projection matrix $\mathbf {P}$:

9 $$\begin{aligned} \mathbf {m}_{{\rm r}}\ =\ \mathbf {P} \ \mathbf {m} , \end{aligned}$$

with the projection matrix, $\mathbf {P}$, obtained from the respiratory subspace as:

10 $$\begin{aligned} \mathbf {P}=\mathbf {V}\left( \mathbf {V}^{T} \mathbf {V}\right) ^{-1} \mathbf {V}^{T}. \end{aligned}$$

As a result, all dynamics of HRV linearly related to respiration are described in $\mathbf {m}_{{\rm r}}$. The orthogonal component, $\mathbf {m}_{\perp }$, computed as the residual, $\mathbf {m}_{\perp } = \mathbf {m} - \mathbf {m}_{{\rm r}}$, is explained by all other HR modulators not linearly related to respiration. An example of the HRV decomposition can be seen in supplementary Figure F2. After decomposing the HRV, the relative power of the respiratory component, ${\mathscr {P}}_{{\rm m}_{{\rm r}}}$, is computed as an estimate of the CPC^38^:

11 $$\begin{aligned} {\mathscr {P}}_{{\rm m}_{{\rm r}}} \ = \ \frac{ \mathbf {m}_{{\rm r}}^{T} \ \mathbf {m}_{{\rm r}} }{ \mathbf {m}^{T}\ \mathbf {m} }. \end{aligned}$$

### Long-term assessment and statistical analysis

Due to the long-term basis of this work, handling missing data is an important issue. First, artefacts, bad detections or ectopic beats appear in the ECG signal. To this end, any NN interval greater than 2.5 s is removed from the series, and the corresponding interpolated segment is also suppressed from the *m*(*t*) signal. Second, regarding the respiratory signal, support MV modes include a backup frequency, and if the patient falls below, the breath cycle is triggered by the ventilator similarly to controlled ventilation. In the cohort study, breathing cycles were labelled as controlled for the 2,11% of them, and were omitted for the analysis. Then, gaps exist in the estimated signals for the CPC calculations. However, results are analysed in 30-min averaged periods for 24 hours, and these gaps or missing data do not affect the results, since they are omitted for the calculation of the average.

At this point, since SDNN is the state-of-art index for medical stratification of cardiac risk in long-term analysis, the SDNN is calculated for the whole period of 24 h before the SBT. The Mann-Whitney U-test is also used to compare the value for the *S-group* vs. *F-group*.

After that, the evolution of the common clinical parameters, HRV and CPC indices, through the 24 h before SBT are analysed, in order to determine if circadian rhythms could be affecting the regulatory mechanisms and the interpretation of the results. However, it must be considered that the SBT’s are generally performed in the morning, but not at the same exact time for all patients. For this reason, all the recordings are segmented from 08:00 p.m. to 10:30 a.m. of the SBT day, so that the same time interval is considered for all patients. This means that it is being considered only some part of the circadian rhythm.

The average value in consecutive 30-min periods is considered, for the representation of the evolution through the day and for the statistical analysis. Table 2 summarizes all the parameters computed to this end. Then, each HRV and CPC indices are calculated separately using different temporal resolutions:

- The parameters F$_r$ and HR are unevenly sampled at the inspiration onset and heart beats occurrence, respectively. Therefore, the mean value in each half hour is computed.
- For the computation of $\mathscr {CE}_{{\rm r} \leftrightarrow {\mathrm{m}}}$, ${\mathscr {P}}_{{\rm m}_{{\rm r}}}$, and the temporal HRV parameters –SDNN and RMSSD–, sliding windows of 3-min-length with 75% of overlap are used. For these, there is a sample each 45 s and a total of 1920 samples in 24 h. So, the mean of 40 overlapped windows in each 30-min period is computed, for each parameter and for each patient.
- The $\mathscr {C}^{\mathcal {T}}_{{\rm HF}}$ and the frequency domain parameters of HRV –${\text{ P }}_{{\rm VLF}}$, ${\text{ P }}_{{\rm HF}}$ and ${\text{ P }}_{{\rm LF}}^n$– are calculated using the TF maps. Therefore, these indices are calculated at the resampling frequency, $F_s=4$ Hz, and thus the average value of 7200 samples, i.e., 30 min, is obtained.

Finally, the averaged values for the *S-group* vs. *F-group* are compared with the non-parametric unpaired Mann-Whitney U-test, for all the parameters. Differences are considered significant for a level of $p \le 0.05$. The effect size Cohen’s *d*, for an acceptable level of statistical power, was also reported. For data where the assumptions of the parametric tests cannot be satisfied, the non-parametric Cohen’s *d* is recommended^50^:

12 $$\begin{aligned} d \ = \ \frac{ Z }{ \sqrt{(n_S + n_F)} }, \end{aligned}$$

where *Z* is the standardized *U*-value, and $n_S$ and $n_F$ are the number of patients of the *S-group* and *F-group*, respectively. A commonly used interpretation is to refer to effect sizes as small ($d=0.2$), medium ($d=0.5$) and large ($d=0.8$).

## Results

**Figure 3:**
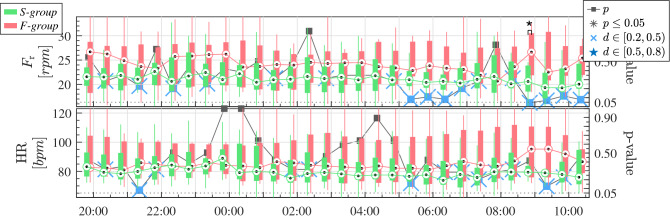
Evolution of the common clinical indices for weaning readiness before SBT. The mean respiratory frequency, F$_r$, and mean HR, are represented. Green and Red boxplots represent the patients of the *S-group* and the *F-group*, respectively. The *p*-value comparing each half hour is represented in the right axis, from 0 to 1, and the dotted line represents the $p=0.05$ threshold. Black asterisks indicate statistical significance with $p \le 0.05$. Blue stars above *p*-values indicate medium effect size with Cohen’s $d \in [0.5,0.8)$, and blue crosses indicate small effect size with Cohen’s $d \in [0.2,0.5)$.

**Figure 4:**
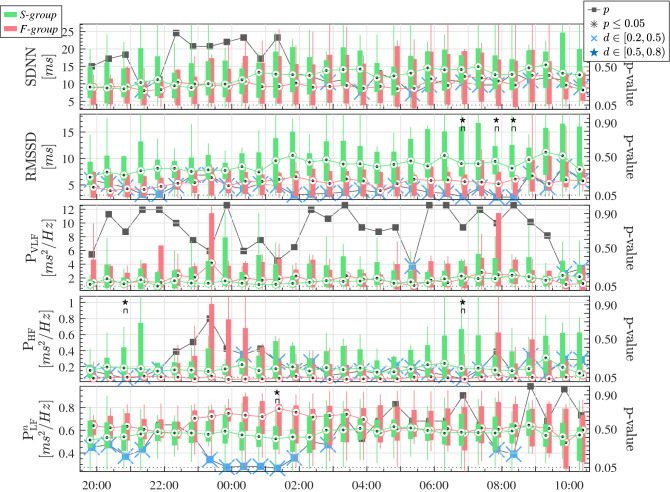
Evolution of the HRV indices before SBT. The temporal parameters SDNN and RMSSD, and the frequency parameters, ${\text{ P }}_{{\rm VLF}}$, ${\text{ P }}_{{\rm HF}}$ and ${\text{ P }}_{{\rm LF}}^n$ are represented. Green and Red boxplots represent the patients of the *S-group* and the *F-group*, respectively. The *p*-value comparing each half hour is represented in the right axis, from 0 to 1, and the dotted line represents the $p=0.05$ threshold. Black asterisks indicate statistical significance with $p \le 0.05$. Blue stars above *p*-values indicate medium effect size with Cohen’s $d \in [0.5,0.8)$, and blue crosses indicate small effect size with Cohen’s $d \in [0.2,0.5)$.

**Figure 5:**
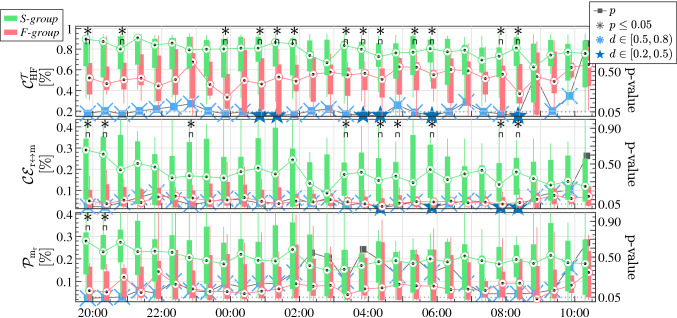
Evolution of the CPC estimators before SBT. The CPC parameters $\mathscr {C}^{\mathcal {T}}_{{\rm HF}}$,$\mathscr {CE}_{{\rm r} \leftrightarrow {\mathrm{m}}}$ and ${\mathscr {P}}_{{\rm m}_{{\rm r}}}$, are represented. Green and Red boxplots represent the patients of the *S-group* and the *F-group*, respectively. The *p*-value comparing each half hour is represented in the right axis, from 0 to 1, and the dotted line represents the $p=0.05$ threshold. Black asterisks indicate statistical significance with $p \le 0.05$. Blue stars above *p*-values indicate medium effect size with Cohen’s $d \in [0.5,0.8)$, and blue crosses indicate small effect size with Cohen’s $d \in [0.2,0.5)$.

Table 3 shows the median and quartiles 1 and 3 of the SDNN, calculated in the whole recordings of 24 h. Higher SDNN is visible for the *S-group* patients, and although the difference is not significant, the *p*-value approaches 0.05. As expected, the SDNN values are higher considering the 24 h recordings (see Table 3), than considering the averaged 3-min windows (see Fig. 4), for the same group of patients. The evolution of the patients throughout the day before SBT, from 08:00 p.m. to 10:30 a.m., are illustrated in the Figs. 3, 4 and 5. The commonly-used clinical variables respiratory frequency, F$_r$, and heart rate, HR—Fig. 3–, can be compared to the parameters of HRV—Fig. 4– and the CPC estimators—Fig. 5.

Looking at Fig. 3, both F$_r$ and HR rely within the limits of criteria for weaning readiness (Supplementary Criteria C1) the whole day. In general, patients of the *F-group* have little higher HR and F$_r$. The F$_r$ is significantly higher only at 9:00, moment when HR differences are larger between both groups. However, no big differences throughout the recordings, during night or day, are appreciable.

**Table 3:** SDNN calculated for the 24 h recordings. Values shown are the inter-subject median and quartiles [$Q_1$, $Q_3$].

		*S-group*	*F-group*	*p*
SDNN	[*ms*]	57 [46–108]	38 [30–58]	0.07

Figure 4 shows the evolution of the HRV parameters. The RMSSD is higher in the *S-group* during the entire recording since midnight, apparently the moment when patients fall asleep. In particular, after waking up, at around 7:00 a.m., significant differences are found. Correspondingly, looking at the ${\text{ P }}_{{\rm LF}}^n$, an increment can be seen for the *F-group*, starting at 00:00, compared to the slight decrease for the *S-group*. This increment for the *F-group* can be associated with a sympathetic activation, in view of the sudden increase of the ${\text{ P }}_{{\rm HF}}$ and ${\text{ P }}_{{\rm VLF}}$ at the very same time.

Curiously, sudden changes can sometimes be found on the ${\text{ P }}_{{\rm VLF}}$, especially for the *F-group*. However, much variability exists for the ${\text{ P }}_{{\rm VLF}}$ power, and neither significant differences nor appreciable patterns on the ${\text{ P }}_{{\rm VLF}}$ evolution can be found.

The evolution of the CPC estimates is illustrated in Fig. 5. As said, CPC estimators are computed considering respiration to be the system driving changes in the HRV. Clear differences exist in the CPC mechanism comparing the patients that were successfully weaned, *S-group*, and the patients reintubated or that still needed time in MV, *F-group*.

The $\mathscr {C}^{\mathcal {T}}_{{\rm HF}}$ index is higher when HRV and respiration have components at the same frequencies, taking into account that these components have different physiological origin. Differences are significantly higher, particularly at night. The $\mathscr {CE}_{{\rm r} \leftrightarrow {\mathrm{m}}}$ is higher during the night than in the morning before the SBT, especially for the *S-group*. The ${\mathscr {P}}_{{\rm m}_{{\rm r}}}$ is also higher for the *S-group* than for the *F-group*. Remark that the *p*-values since 9:00 a.m. approximately, right before the SBT, increases abruptly for the three CPC indices. This shows that the differences between the two groups are less substantial at the time right before performing the SBT. Medium effect size ($d=0.5$) is only present for some comparisons of the CPC parameters (Fig. 5). For the other parameters (Figs. 3, 4), small effect sizes ($d=0.2$) or no effect sizes are observed.

## Discussion

First of all, it should be noted that all patients in the cohort study were deemed ready for weaning. Then, as the SBT determined, some of them were ready, some of them were not, and some of them needed reintubation despite being determined ready by the SBT. Therefore, the prediction of patients ready to undergo SBT has to be improved, in this work for 9 patients: the 2 patients who passed SBT and needed reintubation plus the 7 patients who failed SBT. This suggests that there is some hidden information (e.g., CPC indices) that is not yet taken into account in assessing weaning readiness before SBT.

HRV and CPC have been analysed for a total of 22 patients presumably ready for weaning, in the 24 h before the SBT. Statistical differences have been found comparing patients who needed reintubation or required more time in MV, the so-called *F-group*, and patients with a successful weaning process, *S-group*. These differences are especially appreciable for the parameters estimating the CPC.

The fact that the CPC changes so much with respect to the *S-group* can be related to a more unstable regulatory system. By monitoring this at night, or continuously, clinicians can obtain additional insight of this stability that can help in making the decision to wean a patient from MV. Then, considering the outcome of the SBT, here it is evaluated if the weaning outcome can be predicted before performing the SBT. Nevertheless, this proposal does not pretend to eliminate the SBT, since SBT is necessary. CPC indices are intended to be used in combination with the current weaning readiness criteria (supplementary Criteria C1), to improve the predictive rate of patients ready to undergo the SBT.

Patients of the *S-group* have higher values of SDNN, calculated over 24 h (see Table 3). A major component of SDNN is due to a higher variability and day-night difference of the HR. This shows a better adaptability of the heart to changes, for patients actually ready for weaning, *S-group*.

Remark that the SBT is not performed at the same time for all patients. Hence, in order to have all recordings of the patients aligned in time, some segments had to be omitted at the start and end of the recordings for some patients. The mean respiratory frequency, F$_r$, was always above 9 rpm, i.e., 0.15 Hz. However, for some patients, it was above 24 rpm, i.e., 0.4 Hz (see Fig. 3). The evolution of the currently-used clinical variables, HR and F$_r$, is very similar for both groups. It is clear that these parameters are not giving useful information to predict weaning readiness.

On the contrary, some HRV parameters seem to better discern both groups. The temporal parameter RMSSD has higher values for the *S-group*, in accordance with the fact that this index quantifies parasympathetic modulation of NN intervals driven by respiration and vagal modulations^49^. These modulations of the vagal activity are also quantified by ${\text{ P }}_{{\rm HF}}$. Notice that the ${\text{ P }}_{{\rm HF}}$ is much higher for some patients of the *F-group*, around 11 p.m., and around 8:00 a.m. The rest of the time, mainly during sleep at night, ${\text{ P }}_{{\rm HF}}$ is higher for the *S-group*, in agreement with the results of^20^. The time when ${\text{ P }}_{{\rm HF}}$ is higher for the *F-group*, occurs before going to sleep and waking up. However, the ${\text{ P }}_{{\rm VLF}}$ and ${\text{ P }}_{{\rm LF}}^n$ are also higher, so strong vagal modulations are in conflict with strong sympathetic activations.

Nevertheless, Fig. 4 is striking and summarizes the uncertainty related to the HRV parameters. From these results, the question arises as to what is the validity of HRV indices, since depending on the time of day at which they are measured the results can be totally different. This fact could be attributed to circadian rhythms, having a strong influence on HRV measurements. For example, the ${\text{ P }}_{{\rm LF}}^n$, commonly used as the standard measure of the sympathovagal balance, is not convenient as a reliable weaning readiness predictor. In fact, it has already been proven that ${\text{ P }}_{{\rm LF}}^n$ is not an appropriate measure of the vagal and sympathetic modulations^38,51^. On the contrary, and this is favourable, CPC indices seem more appropriate since measurements are quite stable throughout the 24-hour record.

The sudden increase in the ${\text{ P }}_{{\rm HF}}$ for the *F-group*, that could be interpreted as an increase of the vagal activity, is not present in the CPC parameters. These patients are under MV, and for them the frequency content in the HF band may not contain only respiratory information (see supplementary Figure F2). These HF components can be a consequence of the non-linear effects of respiration transferred to the HR. These non-linear influences could be mediated by the respiratory pacemaker in the central nervous system^52^ through sympathetic modulations^53^, and the CPC estimates used in this work are unable to detect them. Further investigation is required using techniques able to take both linear and non-linear effects into account^54,55^.

At this point, results based on the heart-lung interactions, as measured by CPC indices, were encouraging. First, the TFC exhibited illustrative results. The $\mathscr {C}^{\mathcal {T}}_{{\rm HF}}$, is the CPC estimator which exhibits larger differences between *S-group* and *F-group* patients. These results are also in agreement with those obtained using mutual information, where higher $\mathscr {CE}_{{\rm r} \leftrightarrow {\mathrm{m}}}$ values were found in the *S-group* than in the *F-group*. Finally, there are also visible differences looking at the relative power of respiration, ${\mathscr {P}}_{{\rm m}_{{\rm r}}}$, inserted into the HRV: patients of the *S-group* had a relative power around the 25% of respiration, but those of the *F-group* had it around 5%. Larger effect sizes are obtained for these CPC indices, and most comparisons result in, at least, small effect sizes. These low values of effect size may be associated with the reduced number of patients. Altogether, this illustrates that those patients who are actually ready for weaning, have good levels of CPC and that their ANS is ready to work, in contrast with the patients who did not pass SBT or needed reintubation. Therefore, these CPC estimators are promising as additional indexes to improve the weaning readiness criteria.

Moreover, these differences in the CPC parameters are more evident during sleep than right before the SBT, what could be due to the loop gain. In other words, patients with failed weaning may be experiencing more apnoea events at night, which is directly related to a reduced RSA and higher cardiovascular risk^31,56^. In fact, the parasympathetic activity is well known to be predominant during sleep at night and, consequently, it is in this moment when the CPC mechanism is stronger.

At this moment, clinicians assess whether patients can perform SBT when they are awake in the morning and conscious. Results suggest that probably, it would be better to check patients’ status at night. In fact, less clear differences are found in the morning right before SBT. Maybe, patients generate high levels of stress and anxiety as SBT approaches, and this may alter their biomarkers toward more alert-related values, introducing some physiological bias for interpretation. However, one limitation is that patients in the ICU may not keep similar sleep patterns.

Other works compare the values of the indices obtained right before SBT with the values obtained during and right after SBT and all of them found differences in the respiratory patterns and respiratory variability parameters^57,58^, or in the HRV parameters^19,20,22,59^. Remark that the SBT lasts 30 min, a really stressing stage for the patient, when they are proved to spontaneous breathing. This protocol represents a challenge, and some parameters could show greater statistical differences in that situation than in “basal” or “resting” conditions. That is the very important difference of the present study with the state-of-the-art; here, the indices and evolution of the patients are obtained only before the SBT. The fact that the prediction of weaning readiness can be improved, could only have been revealed by long-term analysis.

From previous data in the literature, variability may be more discriminant when measured during SBT. In fact, it would have been interesting to add values during SBT, although it is a different approach than the proposed. However, the main limitation is that CPC indices cannot be calculated during SBT since patients are disconnected from the ventilator and the respiratory airflow signal is not available. Instead, a surrogate signal such as impedance pneumography, diaphragmatic effort or even an ECG-derived respiratory signal could be used, but this work needs to be developed.

In^60^, they stress the importance of the ${\text{ P }}_{{\rm VLF}}$ power in the weaning scenario. The ${\text{ P }}_{{\rm VLF}}$ power, partially related with the circadian rhythms^49^, could provide useful information of the neurohumoral regulatory mechanism^61^. On the contrary, here it is illustrated that the ${\text{ P }}_{{\rm VLF}}$ power is not so relevant in the analysis of the 24 h prior to SBT. Nevertheless, some patients in this MV and ICU context showed strong characteristic VLF oscillations, possibly also related with sleep disorders, that must be further studied. These sudden changes cause non-stationarity, and this is the reason why 3-min sliding windows and TF analysis are used to calculate the indices.

It should be noted that the estimation of CPC is a new technique, and even less any previous studies have investigated CPC during automated ventilation. Further studies should be performed including controlled MV modes, since this would include the whole amount of patients in MV, and it could be obtained a complete knowledge of the CPC regulation mechanisms. Perhaps, in controlled MV modes, it is the ventilator the one controlling respiration –not the ANS–, and the HRV would be the driving system, since the ventilator would be the one regulating respiration externally.

In this work, the CPC is computed to measure and characterize the RSA function. However, the mechanisms responsible for RSA are still a matter of debate, but it is known to be affected by direct parasympathetic modulation, different reflexes such as baroreflex and chemoreflex, as well as mechanical effect of respiration. Then, there is a clear connection between blood volume and RSA and other predictors could also give estimates of the ANS status. Previous works analysed predictors of fluid responsiveness in mechanically ventilated adults^62^, that could be helpful in this context. In fact, a previous study of us was published analysing the baroreflex for the patients of the cohort study with the blood pressure signal available^63^. Two methods were used to the analyse the baroreflex the hour prior to SBT, and it was found that the capacity of the baroreflex sensitivity was also stronger for patients who were ready for weaning.

The computational cost for obtaining the CPC indices is low, and it may be obtained in real time in the same way as the standard clinical parameters. Therefore, these algorithms can be implemented in the ICU monitors, and the CPC status could be assessed continuously, together with the well-known clinical variables. Additionally, other CPC estimators were also explored in a preliminary analysis of the study. However, the indices with best performance were the ones also computed here, namely ${\mathscr {P}}_{{\rm m}_{{\rm r}}}$, $\mathscr {CE}_{{\rm r} \leftrightarrow {\mathrm{m}}}$ and $\mathscr {C}^{\mathcal {T}}_{{\rm HF}}$. Interestingly, the study in^34^ analyses the best methods for CPC estimation in a simulation study, and concludes that the same three parameters used in this work are the best estimators for CPC assessment.

The clinical utility of this work, and future studies, is that if the CPC is actually proven to predict weaning failure, it might be incorporated as screening guidance of patients ready to undergo SBT. Certainly, there is future work to state how much these CPC indices can improve prediction of weaning readiness. However, this is a preliminary study, which needs to be prospectively validated with a larger cohort. In fact, we still need to report specificity and sensitivity to obtain the thresholds of CPC for which an improvement in accuracy is defined.

In other words, CPC could be assessed before SBT, in a multimodal index combined with current parameters to reinforce the prediction of patients ready to undergo SBT. CPC indices may be of clinical interest since they could help to reduce weaning failure rates and the very adverse effects associated with reintubation, to thereby result in better clinical outcomes.

Finally, it must be kept in mind that patients in the ICU are admitted from very diverse diagnostics. Taking this into account, two patients with the same characteristics and similar evolution may get different outcomes. Hence, sometimes, a patient who does not meet the readiness criteria can be also successfully weaned, and viceversa^64^. This is why clinicians take the criteria for weaning readiness and SBT performance as one among several considerations rather than rigid requirements. In fact, the screening criteria for weaning readiness and SBT are not homogeneous in all sites, and this is a limitation. Such uncertainty can be reduced by implementing new research and technologies to daily clinical practice^65,66^, and this study is other step forward in the field of predictive precision medicine, that exploits the capabilities of the CPC estimates.

## Conclusions

This study analysed the evolution of patients undergoing weaning from MV in the 24 h before SBT, considering that all patients were deemed “ready” for discontinuation from MV. Furthermore, this evaluation was done looking only at the biomarkers before performing the SBT but, because of this, it was needed a long-term analysis. None of the parametric measures currently used as clinical criteria for weaning readiness showed notable differences between patients actually ready for weaning and patients who did not pass the SBT or that needed reintubation. However, it was revealed that patients successfully weaned exhibited relevant higher CPC values, assessed with the variables $\mathscr {C}^{\mathcal {T}}_{{\rm HF}}$, $\mathscr {CE}_{{\rm r} \leftrightarrow {\mathrm{m}}}$ and ${\mathscr {P}}_{{\rm m}_{{\rm r}}}$. In addition, differences were more evident at night. Thanks to the long-term basis of this work, it was also revealed that HRV parameters can be totally different depending on the hour they are measured, and their reliability is questionable to predict weaning readiness.

As stated, the information that clinicians handle in the ICU at the moment is not enough to decide weaning readiness, since there are 15–20% of patients that failed SBT or needed reintubation. By way of conclusion, it was found that the prediction of these failed weaning attempts could be improved if CPC indices were included in the weaning readiness criteria. Then, fewer patients who were not ready for weaning would perform SBT. The potential predictive power of these CPC indices should be considered and further studied, in order to implement this technology into the daily clinical practice. This will help to reduce weaning failure rates, the very adverse effects associated with the reintubation process and thereby result in better clinical outcomes.

## Supplementary Information

Supplementary Information 1.

Supplementary Information 2.

Supplementary Information 3.

Supplementary Information 4.
