# Supplementary material for: Cardiopulmonary coupling indices to assess weaning readiness from mechanical ventilation
Source: Sci Rep. 2021 Aug 6;11:16014. doi: 10.1038/s41598-021-95282-2 (PMC8346488; doi:10.1038/s41598-021-95282-2)
Supplement: Supplementary file 1 — Supplementary Information 1. [file 41598_2021_95282_MOESM1_ESM.pdf]

Supplementary Criteria C1: Weaning Readiness

The following criteria were used to determine if a patient was presumably ready to be weaned, i.e., if a patient was ready to perform the SBT[9–11]:

1. Medical Assessment
    - Improvement or recovery of the cause for MV
    - Adequate cough
    - Absence of secretion
    - No neuromuscular blocking agents

2. Parametric Measures
    - Time in MV > 24 hours
    - $HR < 140$ bpm
    - $SBP \in [90, 160]$ mmHg
    - Haemoglobin (Hb) $\geq$ 8g/dL
    - SpO_2 >90%, with $FiO_2 \leq 40\%$
    - Tidal Volume (V_T) > 5 mL·kg
    - $F_r < 35$ rpm
    - pH $\geq$ 7.30 (no respiratory acidosis)
    - Body Temperature (T) $\in$ [35,38] ºC
    - PEEP $<$ 8 cmH_2O
    - Maximal Inspiratory Pressure (MIP) $\leq$ -25 cmH_2O
    - Rapid Shallow Breathing Index (RSBI) $<$ 105
    - Richmond Agitation-Sedation Scale (RASS): -1/0
    - Glasgow Coma Scale (GCS) >8
    - Minimal vasopressors or inotropes < 5 μgr/kg/minute (stable cardiovascular status)
