# Supplementary material for: Cardiopulmonary coupling indices to assess weaning readiness from mechanical ventilation
Source: Sci Rep. 2021 Aug 6;11:16014. doi: 10.1038/s41598-021-95282-2 (PMC8346488; doi:10.1038/s41598-021-95282-2)
Supplement: Supplementary file 2 — Supplementary Information 2. [file 41598_2021_95282_MOESM2_ESM.pdf]

Supplementary Criteria C2: successful SBT

Once a patient is deemed ready for weaning, they had to perform the SBT. The SBT was carried out by a low-level inspiratory pressure support or by a T-tube test. The criteria listed in this section show the indicators used to evaluate the success of the SBT, in order to decide if MV could finally be withdrawn.

The following criteria were used to assess if SBT was successful[9–11]:

- Good tolerance to the SBT
- $F_r < 35$ rpm
- HR < 140 bpm
- HR $< 20\%$ change from baseline
- $SpO_2 > 90\%$ or $PaO_2 > 60$ mmHg on $FiO_2 < 40\%$
- SBP $\in [80, 180]$ mmHg
- SBP$< 20\%$ change from baseline
- No signs of increased work of breathing or distress
