# Supplementary material for: Cardiopulmonary coupling indices to assess weaning readiness from mechanical ventilation
Source: Sci Rep. 2021 Aug 6;11:16014. doi: 10.1038/s41598-021-95282-2 (PMC8346488; doi:10.1038/s41598-021-95282-2)
Supplement: Supplementary file 3 — Supplementary Information 3. [file 41598_2021_95282_MOESM3_ESM.pdf]

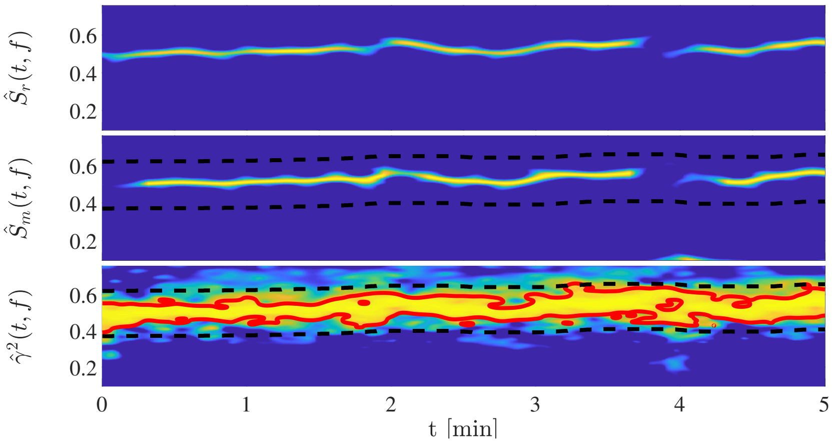

Supplementary Figure 1. TF maps of HRV, respiration and spectral coherence of a five minutes period. The TF map of the respiratory signal, $\hat{S}_r(t,f)$, is on (top) and the TF map of the HRV modulating signal, $\hat{S}_m(t,f)$, is in the (middle). The TF map of the quadratic spectral coherence between respiration and HRV, $\hat{\gamma}^2(t,f)$, is in the (bottom). The black-dotted lines in the TF map of $\hat{S}_m(t,f)$ and $\hat{\gamma}^2(t,f)$, represent the HF band centred at the respiratory frequency, $\Omega^r_{\mathrm{HF}}(t)$. The regions of coherence with statistical significance within the HF band centred at the F_r, $\Omega^{r,c}_{\mathrm{HF}}(t,f)$, are the red-colored area in the $\hat{\gamma}^2(t,f)$ map. For further information, see[36].
