# Supplementary material for: Cardiopulmonary coupling indices to assess weaning readiness from mechanical ventilation
Source: Sci Rep. 2021 Aug 6;11:16014. doi: 10.1038/s41598-021-95282-2 (PMC8346488; doi:10.1038/s41598-021-95282-2)
Supplement: Supplementary file 4 — Supplementary Information 4. [file 41598_2021_95282_MOESM4_ESM.pdf]

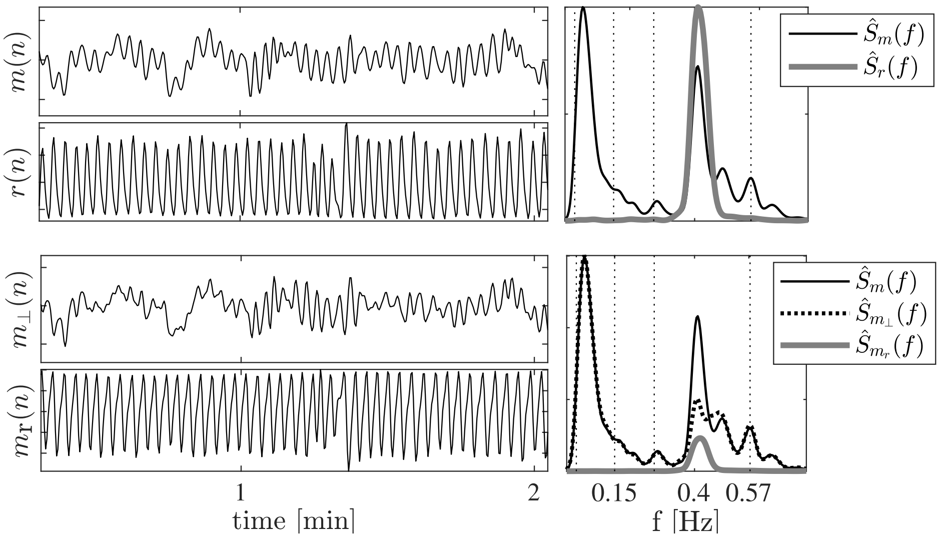

Supplementary Figure 2. Illustrative example of the OSP decomposition using the HRV and Respiratory signals. The three upper plots are the time evolution of the modulating signal, $m(n)$, the respiratory signal, $r(n)$, and their respective spectra on the upper-right side, $\hat{S}_m(f)$ for $m(n)$ and $\hat{S}_r(f)$ for $r(n)$. The three plots below represent the OSP decomposition. The respiratory component of HRV, $m_r(n)$, is obtained projecting $m(n)$ onto the respiratory subspace. The modulators of HRV unrelated to respiration are represented in the term $m_\perp(n)$. Their corresponding spectra are on the lower-right side. $\hat{S}_{m_\perp}(f)$ corresponds to the spectra of $m_\perp(n)$, and $\hat{S}_{m_r}(f)$ to the spectra of $m_r(n)$. For further information, see[38].
